# PD-L1 expression in pleural effusions of pulmonary adenocarcinoma and survival prediction: a controlled study by pleural biopsy

**DOI:** 10.1038/s41598-018-29156-5

**Published:** 2018-07-25

**Authors:** Jian Xu, Xue Han, Chunfang Liu, Na Gao, Junjun Zhao, Xiaolin Zhang, Ling Jiang, Lina Ren, Ping Li, Nini Wang

**Affiliations:** 1 Department of Respiratory, Dalian Municipal Central Hospital affiliated with Dalian Medical University, Dalian, 116033 China; 2 Department of Pathology, Dalian Municipal Central Hospital affiliated with Dalian Medical University, Number 826, XiNan Road, Dalian City, 116033 Liaoning Province China

## Abstract

PD-L1 expression in pleural effusions (PE) of lung adenocarcinoma (ADC) was compared with pleural biopsies and the positive expression in PE was correlated with survival time. The matched slices from same patient’s pleura and PE were collected which both were pathologically verified positive. Immunohistochemistry (IHC) was used to detect PD-L1 expression. A total of 51 eligible cases were enrolled, including 30 males and 21 females. The average age was (67.06 ± 12.10) years. PD-L1 expression wasn’t statistically significant in pleura and cell masses (P > 0.05) and the correlation was statistically significant (r = 0.585, P = 0.000). Using an IHC scores of 5 point as a cutoff, positive PD-L1 expression in the pleura was 11.63% and that in the cell masses was 23.26%, and difference was significant (P < 0.05). The correlation coefficient was 0.605. Among 35 cases underwent systemic anti-tumor treatment, the mean survival time with positive PD-L1 expression in PE was 17.370 ± 1.827 months, which was significantly shorter than that with the negative (29.944 ± 2.671 months) (χ^2^ = 4.507, P = 0.034). Positive PD-L1 expression in PE is higher than in the pleura and their correlation is well. It may predict the short survival time after systemic anti-tumor treatment.

## Introduction

Non-small-cell lung cancer (NSCLC) is the most common cause of cancer-related death worldwide. More than 65% of NSCLC present with locally advanced or metastatic disease^1^. These patients are treated with chemotherapy or tyrosine kinase inhibitors as a first-line. Recently, anticancer immunotherapy targeting immune checkpoints with antibodies against programmed death-1 (PD-1) and its ligand PD-L1 has emerged as a promising therapeutic strategy^2^. PD-1 is expressed on tumor antigen-specific T cells and cancer cells. PD-1 receptor has two ligands: PD-L1 and PD-L2. PD-L1 is expressed on tumor cells, T cells and monocytes/macrophages. Binding of PD-1 and PD-L1 inhibits activation of T cells and allows tumor cells to bypass immune surveillance. Inhibition of this pathway with PD-1 inhibitors, such as nivolumab, releases effector immune T cells and allows for their anti-tumor action^3^. Several ongoing international clinical trials demonstrate significant better overall survival (OS) in PD-1 or PD-L1 inhibitor than in docetaxel combined cisplatin. However, the response rate to PD-1 or PD-L1 blockade is approximately 20%, indicating the need for a predictive biomarker^4^. PD-L1 protein expression assessed by immunochemistry has emerged as a biomarker to select patients and predict anticancer immunotherapy^5,6^. The expression of PD-L1 is also correlated with the clinicopathologic features and prognosis of platinum based chemotherapy^7^. The conflicting results call for more studies to achieve a consensus before it can be used routinely in the clinic. Meanwhile, little is known about the consistency of PD-L1 expression in pleural effusions (PE) and pleural tissue. PE often occurs during advanced lung cancers, which are treated with anticancer immunotherapy. The cells in effusions exist in a different environment, which may have an effect on morphological features as well as the expression of various biomarkers^8^. Therefore, investigation of PD-L1 expression in PE may be necessary and feasible for treatment outcome and survival predictor. In this study, we detect and compare the differences of PD-L1 expression between matched PE and pleural tissue in stage IV pulmonary adenocarcinoma (ADC) and evaluate the correlation of PD-L1 expression in PE with survival time after antitumor therapy.

## Results

### Clinical data

The clinical data of the eligible cases are shown in Table 1. Patients were following up until December 2017, and 30 of them had defined outcomes, 19 were surviving, and 2 were missing.

**Table 1:** Clinicopathologic parameters of patients.

Clinicopathologic parameter	N (total = 51)
Median age	67.06 ± 12.10
Sex	
Male	30
Female	21
Smoking	
Never	39
Ever	12
Stage IV	51
Distant metastasis	25
Brain	15
Bone	5
Live	2
More than two distant organs	3
EGFR mutation	20
Anticancer therapy	
No	8
Intrapleural injection of cisplatin	8
Systemic chemotherapy	15
EGFR-TKIS	20

### The consistency of PD-L1 expression in pleura and PE

**Figure 1:**
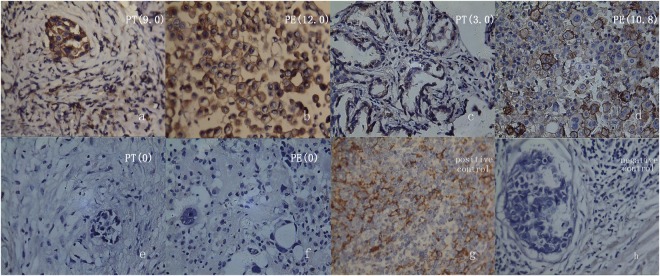
PD-L1 expression in pleural tissue (PT), pleural effusions (PE) of pulmonary adenocarcinoma by immuhistochemical stain (400 times magnification). The slices of (**a**–**f**) are matched respectively by PT and PE from the same patients. If 5 points is the cut-off value, PD-L1 expression of PT and PE are both positive in the first case, a negative and a positive in the second case, both negative in the third case. The (**g** and **h**) are positive (lymphoma) and negative (breast carcinoma) control. The values in brackets are the mean scores in each slice of five scopes.

The mean IHC scores were 2.506 ± 2.526 and 3.141 ± 3.603 respectively in pleura and PE, the difference wasn’t statistically significance (t = −1.537, P = 0.131), and their correlation was statistical significant (r = 0.585, P = 0.000). If the IHC scores of 1, 3 and 5 points were selected as the cutoff value to define the positive PD-L1 expression, the positive expression and statistical significance were listed in Table 2. If 5 points was the cutoff value, the positive PD-L1 expression in PE was significantly higher than that in the pleura, they were 23.26% vs 11.63% respectively, the difference was statistically significant (P < 0.05). Their correlation was well [Fig. 1], and the correlation coefficient was 0.605.

**Table 2:** PD-L1 expression depending on different cutoff values in pleural tissue and PE.

cutoff value	pleural % (cases)	cell block % (cases)	χ^2^ value	P value	κappa
1	64.71(33/51)	64.71(33/51)	0.878	0.349	0.227
3	35.29(18/51)	41.18(21/51)	0.298	0.585	0.131
≥5	11.76(6/51)	23.53(12/51)	7.367	***0.007***	0.605

There is statistically significant difference when P <0.05.

### PD-L1 expression and survival time

Among 51 eligible cases, 35 cases undergone systemic anti-tumor therapy included systemic chemotherapy and/or EGFR-TKIs, and 16 cases didn’t have therapy or only have intrapleural injection of cisplatin (Table 1). The mean survival time was 8.813 ± 3.534 months (95% CI: 1.885–15.740) in cases without systemic anti-tumor therapy, and it was 27.284 ± 2.443 months (95%CI: 22.496–32.073) in cases under systemic anti-tumor therapy. The difference of survival time was statistically significant (χ^2^ = 21.010, P = 0.000, P < 0.05).

Among patients with systemic anti-tumor therapy, PD-L1 expression and survival time were listed in Table 3. The mean survival time was significantly shorter in PD-L1 positive expression than the negative expression depending on more than IHC scores of 5 points in PE (P < 0.05). This might indicate that positive PD-L1 expression had the poor systemic anti-tumor therapeutic effects. The survival time wasn’t done depending on more than 5 points in the pleura because all of 6 cases were survived.

**Table 3:** The mean survival time and positive expression of PD-L1 in patients under anticancer therapy.

cutoff value	specimen	PD-L1 expression	mean survival time (month)	standard deviation (month)	95% confidence interval		χ^2^ value	P value
					lower	upper		
1	pleural	−	26.727	3.284	20.291	33.164	0.415	0.519
		+	28.634	3.700	21.244	36.023		
	cell	−	30.750	3.859	23.187	38.313	2.134	0.144
	block	+	21.528	1.692	18.212	24.843		
3	pleural	−	26.850	2.737	21.485	32.215	0.028	0.868
		+	20.200	1.953	16.371	24.029		
	cell	−	30.426	2.999	24.548	36.304	2.912	0.088
	block	+	19.727	2.197	15.422	24.033		
5	pleural	−						
		+						
	cell	−	29.944	2.671	24.710	35.179	4.507	***0.034***
	block	+	17.370	1.827	13.789	20.951		

There is statistically significant difference when P < 0.05.

## Discussion

Checkpoint inhibitor immunotherapy has become the most exciting topic in oncology. In NSCLC, the higher response rate and the lower toxicity make immunotherapy a very attractive alternative therapy compared to chemotherapy^9^. Future goals and hopes are finding ways to predict who will respond to immunotherapy. Even though PD-L1 has been the most studied biomarker, it has not been fully validated due to the lack of definition of the threshold for positive PD-L1 labeling on tissue samples. Different studies selected different cutoff values to define expression of PD-L1. Roach *et al*.^4^ investigated the correlation of PD-L1 status with the clinical outcome in a phase 1 clinical trial (KEYNOTE-001), and found that PD-L1 expression in more than 50% of tumor cells correlated with 41% overall response rate to pembrolizumab. Shien *et al*.^10^ noted that those with more than 5% PD-L1 expression on tumor cells had a higher response rate than those with negative PD-L1 during nivolumab treatment. And some others evaluated the positive PD-L1 expression depending on different H-score of staining tumor cells^11^.

Then, studies found that PD-L1 expression depended on tumor type, differentiation and advanced stages. Shimoji *et al*.^11^ confirmed the significant higher positivity of PD-L1 expression in squamous cell carcinoma (SCC) than in ADC. But, Ohue *et al*.^12^ indicated that it was the opposite. Sun *et al*.^13^ confirmed a significantly higher prevalence of PD-L1 positivity among SCC, stage IIIB and IV lung cancer. Yeo *et al*.^14^ observed the higher expression of PD-L1 with poor differentiated subtype in ADC and advanced stage in SCC.

Some researchers also evaluated the concordance of PD-L1 expression between primary tumor and metastatic lesions. Kim and Uruga *et al*.^3,15^ paired primary and metastatic tumor tissues in the resected ADC and confirmed overall concordance rate for PD-L1 expression. Ameratunga *et al*.^16^ matched tumor and nodal specimens in NSCLC and noted highly concordant for PD-L1 expression. Ilie *et al*.^17^ matched preoperative biopsy and surgical resection specimens and noted moderate concordance for high PD-L1 scoring groups. These studies indicated that small biopsy specimens from metastatic lesion were feasible for detecting PD-L1 expression and PD-L1 expression was concordant among paired primary and metastatic tumor.

In this study, we detected and compared PD-L1 expression in pleural biopsy and PE from same patients to avoid interference of tumor histological types and clinical stages by limiting all eligible cases to stage IV pulmonary adenocarcinoma. The IHC scores of 5 points in this paper meant that tumor cells were stained more than 50% with medium membranous staining or more than 25% with sepia staining. This was similar to 50% as the cutoff value in the other studies. Using this cutoff, positive PD-L1 expression was significantly higher in pleural effusions than pleural biopsies. The causes were supposed to tumor heterogeneity and the tumor cells in pleural effusions coming from more lesions than biopsies. Heymann and colleague’s finding^18^ was in line with our results, they confirmed that cytological specimens were more likely to be positive compared with histological specimens.

Studies^19,20^ showed that PD-L1 expression might be the predictive marker associated with higher ORR, longer PFS and OS in patients with checkpoint inhibitor immunotherapy. However, studies revealed conflicting results on PD-L1 expression and survival prediction in patients without immunotherapy. He *et al*.^21^ showed that patients with positive PD-L1 expression on tumor cells had shorter relapse-free survival (RFS) than those with negative PD-L1 expression. Paulsen *et al*.^22^ evaluated disease-specific survival (DSS) and reported that low tumor epithelial expression of PD-L1 had a significant negative prognostic impact on DSS in SCC patients, while PD-L1 status was not associated with survival for patients with large cell carcinoma or ADC. Yeo *et al*.^14^ noted that positive PD-L1 expression was a prognostic factor which indicated a shorter disease-free survival (DFS) in ADC. Igawa *et al*.^7^ indicated that the 5-year cumulative survival probability for patients with high PD-L1 expression was lower but not significant in SCC. Okita^23^ and Wu^24^ observed that PD-L1 over-expression was associated with poor RFS and OS. Sun^14^ associated PD-L1 expression with poorer OS and progression-free survival (PFS). Mu *et al*.^25^ observed significantly worse overall 3-year survival in patients with PD-L1 positive tumors. Several meta-analyses^26,27^ have shown that high PD-L1 expression was also correlated with poor prognosis in terms of the OS of patients with NSCLC. But, Cooper and colleagues^28^ observed that patients with high PD-L1 expression had significantly longer OS in early stage of lung cancer. In this study, positive PD-L1 expression in PE was significantly correlated with shorter survival times in patients under systemic anti-tumor therapy and without checkpoint inhibitor immunotherapy. This was in consistent with the most of studies above-mentioned. More researches should be explored owing to only 51 cases involved.

It was feasible to detect PD-L1 expression in pleural effusions in advanced ADC. The positive PD-L1 expression in pleural effusions was higher than in pleural tissues, and their consistency was well. It might predict the short survival time after systemic anti-tumor treatment.

## Methods

### Cases collection

We collected lung adenocarcinomas from January 2013 to December 2016 at the Department of Respiratory, Dalian Municipal Central Hospital, which was confirmed by thoracoscopy and whose pathology in the intraoperative pleural effusion cell block was also positive. Approval for the study was obtained from the Hospital Human Ethics Committee and all methods involved in this study were performed in accordance with Declaration of Helsinki. All participants had signed the informed consent form.

Medical thoracoscopy was performed under local anesthesia and the procedure followed the instruction of manufacturer (LTF 240, Olympus, Japan). 200 ml pleural effusion was collected during operation. All PE were centrifugated for 5 minutes at 2000 r/min and the supernatant fluid was removed. The pellet was fixed directly in 10% formalin before paraffin embedding. Cell blocks and pleural tissues of eligible patients were inspected uniformly. Immunohistochemistry (IHC) was used to detect the expression of PD-L1 in the cell mass and pleural tissue sections, and the difference of PD-L1 expression in the two specimens was compared. The patient’s clinical stage, treatment plan, and survival time were recorded, and the correlation between PD-L1 expression and survival was assessed.

### Immunohistochemical staining procedure

Paired paraffin sections of 4 μm from same case’s pleura and pleural effusion cell block were placed on the same slide, dewaxed, 9% sodium citrate buffer for antigen retrieval, and rabbit anti-human PD-L1 monoclonal antibody (Abcam, clone ZR3, UK) with 1:200 dilution of the primary antibody was applied incubation at room temperature for an hour. We added the rat/rabbit universal secondary antibody (Fuzhou Maixin Co., China) incubated at room temperature, plus DAB color reagent, hematoxylin counterstained, neutralization of resin sealing after the slices were dried.

The expression of PD-L1 in each slice was read independently by two pathologists. The results were analyzed using semi-quantitative methods. The positive cells were the brownish yellow coloration of the tumor cell membrane. Each section was randomly selected five high-magnification fields of view (400 times), and the total number of tumor cells and the number of stained positive cells in each field were counted. Positive staining of tumor cells was < 10% for 0 points, $\geqslant$10% and <25% for 1 point, $\geqslant$25% and <50% for 2 points, $\geqslant$50% and <75% for 3 points, $\geqslant$75% for 4 points. The staining intensity score was based on the staining state exhibited by most cells, with no coloration being 0 points, light yellow being 1 point, brownish yellow being 2 points, and sepia being 3 points. The product of the staining intensity score and the positive cell ratio score was the final scores (IHC scores).

### Statistical analysis

SPSS22.0 statistical software was used. Differences in PD-L1 expression scores between pleural effusions and pleura were analyzed using t-tests with two relevant samples. Results were expressed as mean ± standard deviation. Differences in the positive detection rate of PD-L1 expression in the two groups of specimens were performed using the χ^2^ test of two related samples. The consistency of PD-L1 expression in the two groups was tested using the Pearson product-moment correlation coefficient of two-variable correlation analysis and the Kappa correlation coefficient of two correlation variables. The total survival time was calculated using log rank survival analysis.

## References

[1] Aguiar PN (2016). A pooled analysis of nivolumab for the treatment of advanced non-small-cell lung cancer and the role of PD-L1 as a predictive biomarker. Immunotherapy..

[2] Wang C, Yu X, Wang W (2016). A meta-analysis of efficacy and safety of antibodies targeting PD-1/PD-L1 in treatment of advanced non-small cell lung cancer. Medicine (Baltimore)..

[3] Uruga H (2017). Programmed Cell Death Ligand (PD-L1) Expression in Stage II and III Lung Adenocarcinomas and Nodal Metastases. J. Thorac Oncol..

[4] Roach C (2016). Development of a Companion Diagnostic PD-L1 Immunohistochemistry Assay for Pembrolizumab Therapy in Non-Small-cell Lung Cancer. Appl Immunohistochem Mol Morphol..

[5] Thakur MK, Gadgeel SM (2016). Predictive and Prognostic Biomarkers in Non-Small Cell Lung Cancer. Semin Respir Crit Care Med..

[6] Shukuya T, Carbone DP (2016). Predictive Markers for the Efficacy of Anti-PD-1/PD-L1 Antibodies in Lung Cancer. J. Thorac Oncol..

[7] Igawa S (2017). Impact of PD-L1 Expression in Patients with Surgically Resected Non-Small-Cell Lung Cancer. Oncology..

[8] Mansour MSI (2017). Determination of PD-L1 expression in effusions from mesothelioma by immuno-cytochemical staining. Cancer Cytopathol..

[9] Sun X (2017). Immune-related adverse events associated with PD-1 and PD-L1 inhibitors for nonsmall cell lung cancer: Protocol for a systematic review and meta-analysis. Medicine (Baltimore)..

[10] Shien K, Papadimitrakopoulou VA, Wistuba II (2016). Predictive biomarkers of response to PD-1/PD-L1 immune checkpoint inhibitors in non-small cell lung cancer. Lung Cancer..

[11] Shimoji M (2016). Clinical and pathologic features of lung cancer expressing programmed cell death ligand 1 (PD-L1). Lung Cancer..

[12] Ohue Y (2016). Survival of Lung Adenocarcinoma Patients Predicted from Expression of PD-L1, Galectin-9, and XAGE1 (GAGED2a) on Tumor Cells and Tumor-Infiltrating T Cells. Cancer Immunol Res..

[13] Sun JM (2016). Prognostic Significance of PD-L1 in Patients with Non-Small Cell Lung Cancer: A Large Cohort Study of Surgically Resected Cases. J. Thorac Oncol..

[14] Yeo MK (2017). Association of PD-L1 expression and PD-L1 gene polymorphism with poor prognosis in lung adenocarcinoma and squamous cell carcinoma. Hum Pathol..

[15] Kim S (2017). Comparative analysis of PD-L1 expression between primary and metastatic pulmonary adenocarcinomas. Eur J Cancer..

[16] Ameratunga M (2016). PD-L1 and Tumor Infiltrating Lymphocytes as Prognostic Markers in Resected NSCLC. PLoS One..

[17] Ilie M (2016). Comparative study of the PD-L1 status between surgically resected specimens and matched biopsies of NSCLC patients reveal major discordances: a potential issue for anti-PD-L1 therapeutic strategies. Ann Oncol..

[18] Heymann JJ (2017). PD-L1 expression in non-small cell lung carcinoma: Comparison among cytology, small biopsy, and surgical resection specimens. Cancer Cytopathol..

[19] Nishio M (2017). Multicentre phase II study of nivolumab in Japanese patients with advanced or recurrent non-squamous non-small cell lung cancer. ESMO Open..

[20] Aguiar PNJ, De MRA, Hall P, Tadokoro H, Lima Lopes G (2017). PD-L1 expression as a predictive biomarker in advanced non-small-cell lung cancer: updated survival data. Immunotherapy..

[21] He Y (2017). PD-1, PD-L1 Protein Expression in Non-Small Cell Lung Cancer and Their Relationship with Tumor-Infiltrating Lymphocytes. Med Sci Monit..

[22] Paulsen EE (2017). Assessing PDL-1 and PD-1 in Non-Small Cell Lung Cancer: A Novel Immunoscore Approach. Clin Lung Cancer..

[23] Okita R (2017). PD-L1 overexpression is partially regulated by EGFR/HER2 signaling and associated with poor prognosis in patients with non-small-cell lung cancer. Cancer Immunol Immunother..

[24] Wu S (2017). The significance of programmed cell death ligand 1 expression in resected lung adenocarcinoma. Oncotarget..

[25] Mu CY, Huang JA, Chen Y, Chen C, Zhang XG (2011). High expression of PD-L1 in lung cancer may contribute to poor prognosis and tumor cells immune escape through suppressing tumor infiltrating dendritic cells maturation. Med Oncol..

[26] Wang A (2015). The prognostic value of PD-L1 expression for non-small cell lung cancer patients: a meta-analysis. Eur J Surg Oncol..

[27] Xia H (2017). PD-L1 over-expression is associated with a poor prognosis in Asian non-small cell lung cancer patients. Clin Chim Acta..

[28] Cooper WA (2015). PD-L1 expression is a favorable prognostic factor in early stage non-small cell carcinoma. Lung Cancer..

